# Supplementary material for: Synthesis of a New Phosphonate-Based Sorbent and Characterization of Its Interactions with Lanthanum (III) and Terbium (III)
Source: Polymers (Basel). 2021 May 8;13(9):1513. doi: 10.3390/polym13091513 (PMC8125837; doi:10.3390/polym13091513)
Supplement: Supplementary file 1 [file polymers-13-01513-s001.zip › polymers-1202753-SI.pdf]

Supplementary Information

Synthesis of a new phosphonate-based sorbent and characterization of its interactions with lanthanum (III) and terbium (III)

Yuezhou Wei [1,2], Khalid A.M. Salih [1], Mohammed F. Hamza [1,3*], Toyohisa Fujita [1], Enrique Rodríguez Castellón [4*] and Eric Guibal [5]

[1] Guangxi Key Laboratory of Processing for Non-ferrous Metals and Featured Materials, School of Resources, Environment and Materials, Guangxi University, Nanning 530004, PR China; yzwei@gxu.edu.cn (Y.W.); Immortaltiger7@gmail.com (K.A.M.S.); m_fouda21@hotmail.com (M.F.H.); fujitatoyohisa@gxu.edu.cn (T.F.)

[2] Guangdong Institute of Rare Metals, Guangdong Academy of Science, Guangzhou, China; yzwei@gxu.edu.cn (Y.W.)

[3] Nuclear Materials Authority, POB 530, El-Maadi, Cairo, Egypt; m_fouda21@hotmail.com (M.F.H.)

[4] Universidad de Malaga, Departamento de Química Inorgánica, Facultad de Ciencias, Malaga, Spain; castellon@uma.es (E.R.C.)

[5] IMT – Mines Ales, Polymers Composites & Hybrids (PCH), F-30319 Alès cedex, France; eric.guibal@mines-ales.fr (E.G.)

* Correspondence: castellon@uma.es; Tel.: +34645263909 (E.R.C.) & m_fouda21@hotmail.com Tel: +202-01116681228 (M.F.H)

Table 1. Optical micrographs for ALPEI, Cl-ALPEI, POH-ALPEI (and average bead size).

Sample	Optical micrograph	Average size
**ALPEI**	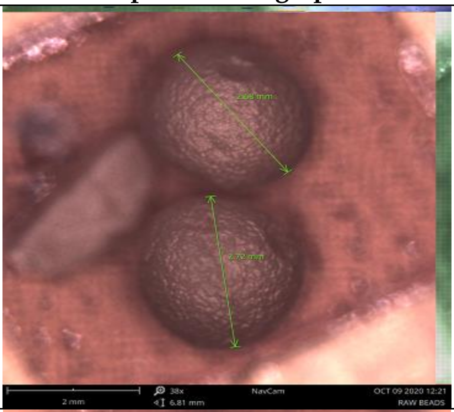	2.7 ±0.02 mm
**Cl-ALPEI**	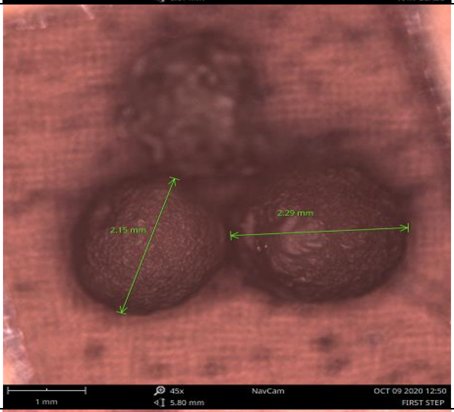	2.195 ±0.07 mm
**POH-ALPEI**	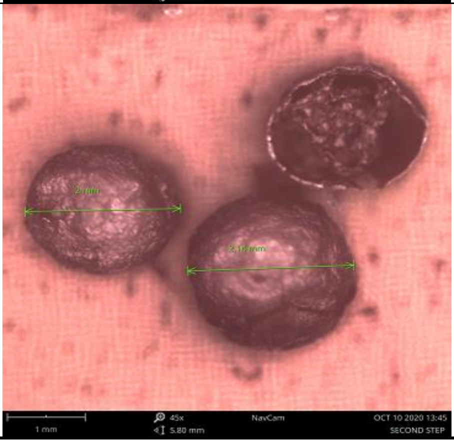	2.07 ±0.07 mm

Table S2. SEM micrographs of ALPEI, Cl-ALPEI, P-ALPEI and POH-ALPEI.

Sample	SEM	Crosscut
**ALPEI**	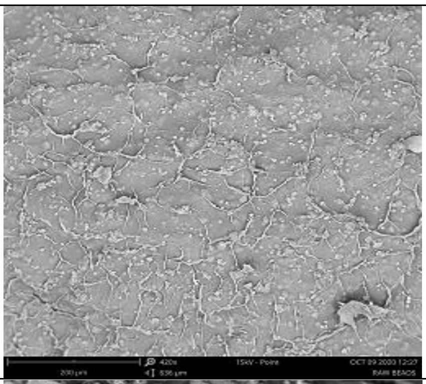	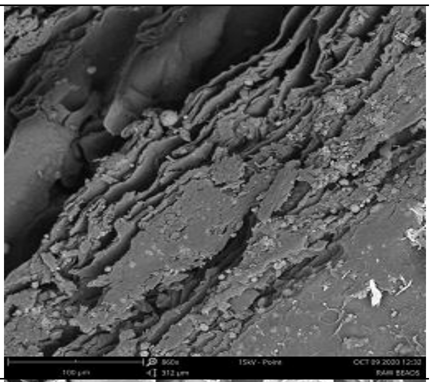
**Cl-ALPEI**	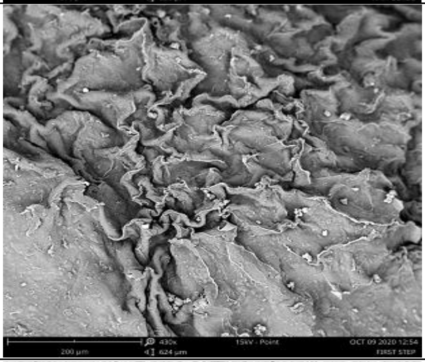	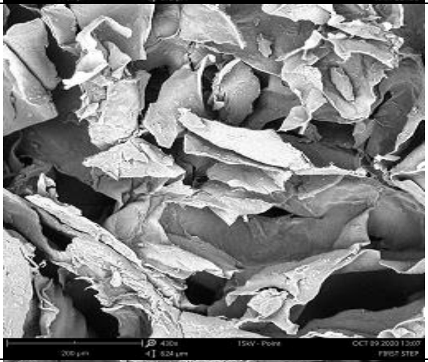
**P*-ALPEI**	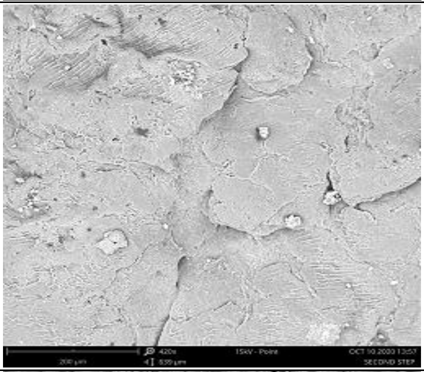	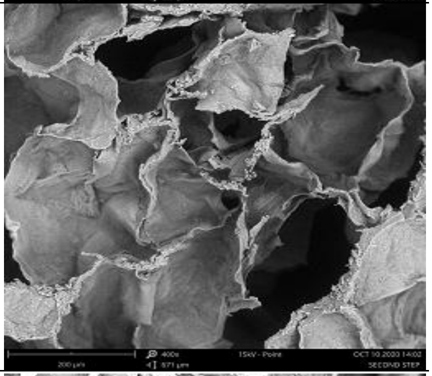
**POH-ALPEI**	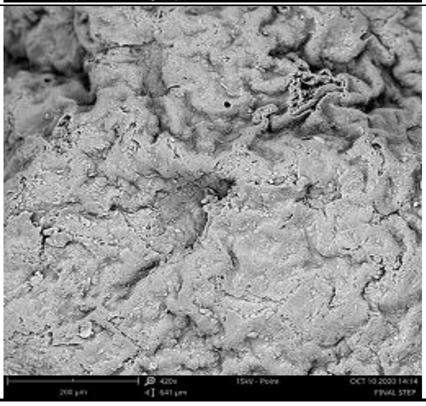	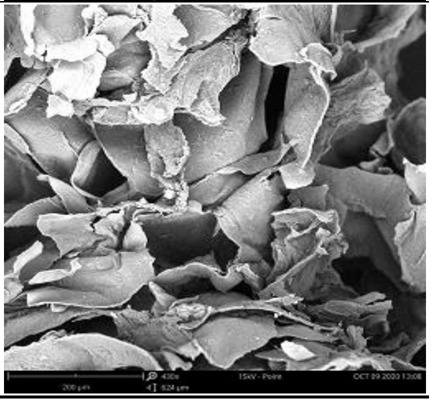

Table 3. Semi-quantitative EDX analysis of surface and crosscut section of ALPEI, Cl-ALPEI, P-ALPEI and POH-ALPEI.

SEM	Crosscut

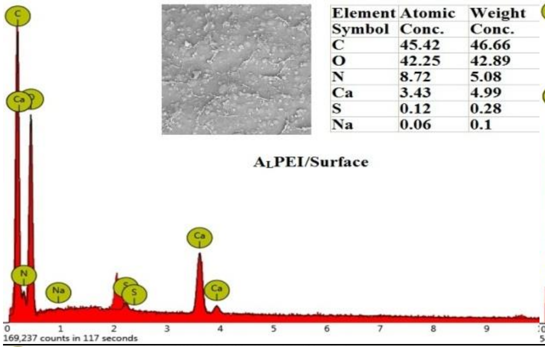

Element Symbol	Atomic Conc.	Weight Conc.
C	45.42	46.66
O	42.25	42.89
N	8.72	5.08
Ca	3.43	4.99
S	0.12	0.28
Na	0.06	0.1

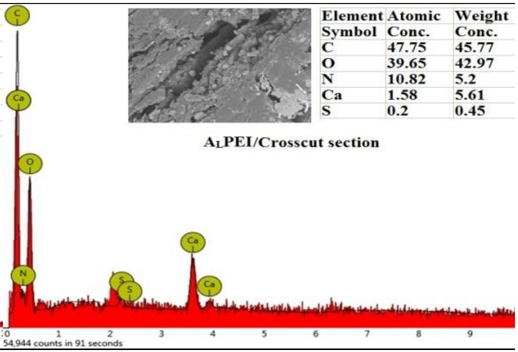

Element Symbol	Atomic Conc.	Weight Conc.
C	47.75	45.77
O	39.65	42.97
N	10.82	5.2
Ca	1.58	5.61
S	0.2	0.45

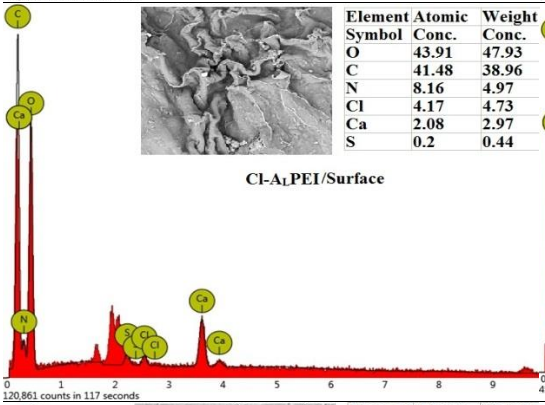

Element Symbol	Atomic Conc.	Weight Conc.
O	43.91	47.93
C	41.48	38.96
N	8.16	4.97
Cl	4.17	4.73
Ca	2.08	2.97
S	0.2	0.44

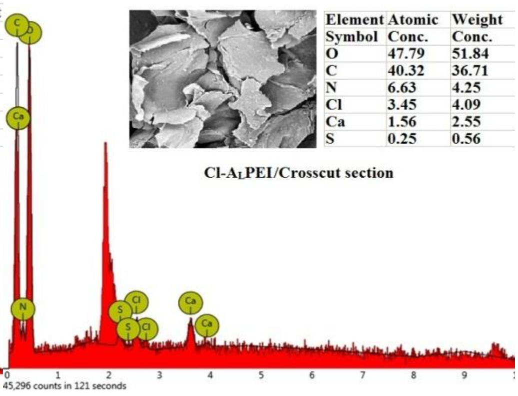

Element Symbol	Atomic Conc.	Weight Conc.
O	47.79	51.84
C	40.32	36.71
N	6.63	4.25
Cl	3.45	4.09
Ca	1.56	2.55
S	0.25	0.56

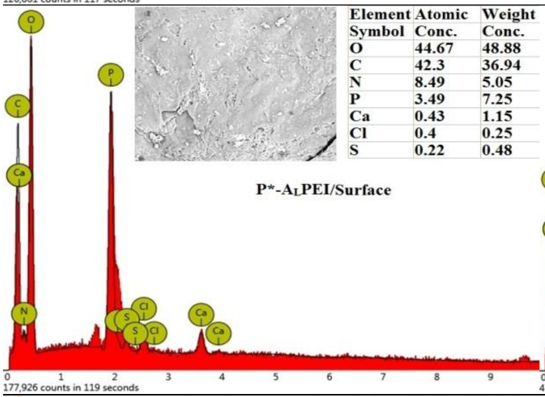

Element Symbol	Atomic Conc.	Weight Conc.
O	44.67	48.88
C	42.3	36.94
N	8.49	5.05
P	3.49	7.25
Ca	0.43	1.15
Cl	0.4	0.25
S	0.22	0.48

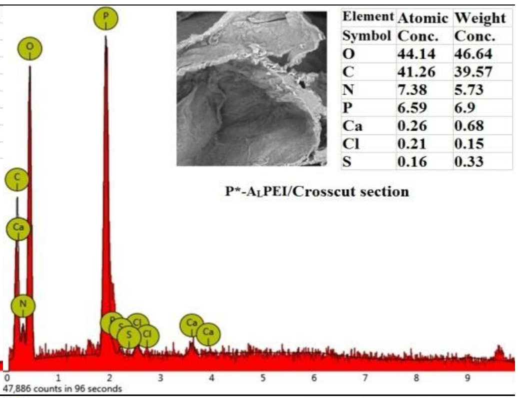

Element Symbol	Atomic Conc.	Weight Conc.
O	44.14	46.64
C	41.26	39.57
N	7.38	5.73
P	6.59	6.9
Ca	0.26	0.68
Cl	0.21	0.15
S	0.16	0.33

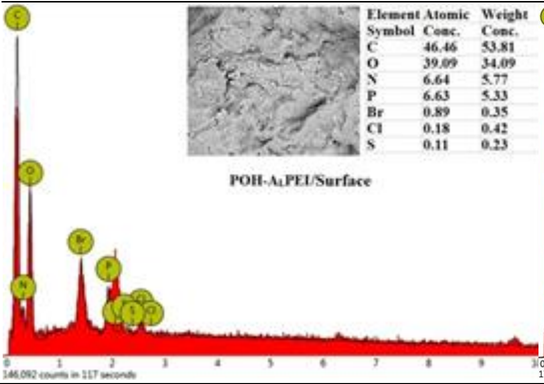

Element Symbol	Atomic Conc.	Weight Conc.
C	46.46	53.81
O	39.09	34.09
N	6.64	5.77
P	6.63	5.33
Br	0.89	0.35
Cl	0.18	0.42
S	0.11	0.23

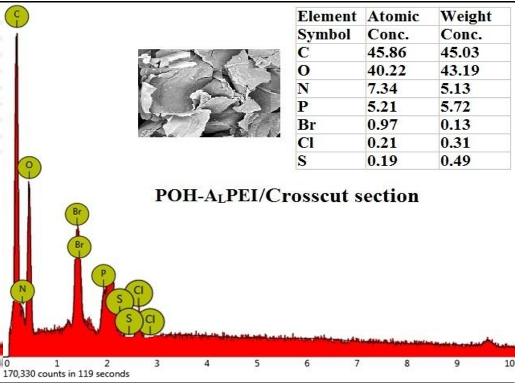

Element Symbol	Atomic Conc.	Weight Conc.
C	45.86	45.03
O	40.22	43.19
N	7.34	5.13
P	5.21	5.72
Br	0.97	0.13
Cl	0.21	0.31
S	0.19	0.49

Table 4. Elemental analysis of ALPEI beads, intermediary products (epichlorohydrine-activated ALPEI, Cl-ALPEI, and phosphorylated ALPEI beads, P*-ALPEI), and sorbent (de-esterified phosphorylated beads, POH-ALPEI).

Material	C (%)	N (%)	N[(a)]	H (%)	O (%)	O[(a)]	S (%)	P (%)	P[(a)]
ALPEI	38.1	4.9	3.48	5.89	34.9	21.80	0.2	0.0	0.00
Cl-ALPEI	40.4	4.1	2.96	6.03	37.6	23.52	0.2	0.0	0.00
P*-ALPEI	41.4	3.9	2.79	6.41	40.5	25.34	0.1	6.5	2.10
POH-ALPEI	39.1	3.9	2.78	6.13	43.2	27.03	0.1	6.8	2.20

(a): molar units for element content (i.e., mmol Element g^{-1}).

Table 5. FTIR assignments and wavenumbers (cm^{-1}) of ALPEI, intermediary products (epichlorohydrine-activated ALPEI, Cl-ALPEI, and phosphorylated ALPEI beads, P*-ALPEI), and sorbent (de-esterified phosphorylated beads, POH-ALPEI).

Vibration	Ref.	Wn. in ref.	ALPEI	Cl- ALPEI	P*-ALPEI	POH- ALPEI
**O-H overlapped with N-H stretch**	**[1, 2]**	**3500 -3000**	**3402**	**3383**	**3477**	3371
Aliphatic C-H (stretch)	[2]	2970-2750	2937/2775	2937/2767	2935/2767	2939/2767
C=O ester (stretch)	[2]	1750-1725	1753	1753	1751	1753
C=O amide (stretch)/ 1° and 2° amine bend (overlapped)	[1, 3, 4]	1690-1630	1620	1622	1631	1622
C-H/ 1°/2° hydroxyl groups bend and $-COO^-$ salt	[1, 5-7]	1485–1260	1382	1381/1213	1381 (broad)	1382 (broad)
Asymmetric P=O	[8-11]	1350-1250				
Phosphate ion P(O) (stretch)	[12, 13]	1100-990				
C-C, C-O and C-N (stretch)	[2, 14-17]	1350–1000	1033	1055/949	1076	1055
OH (bend) out-of-plane	[1, 2]	750–590	833			
CH_2-Cl	[18]	700-800		808		
P-O-C band (stretch)	[8-12]	570/1100-990			802/526	810/570

Table S6. Assignments and interpretation of major XPS signals for ALPEI, intermediary products (i.e., Cl-ALPEI and P*-ALPEI) and P2-ALPEI.

**Signal**	ALPEI		Cl-ALPEI		P*-ALPEI		POH-ALPEI		Assignment
	BE (eV)	AF (%)	BE (eV)	AF (%)	BE (eV)	AF (%)	BE (eV)	AF (%)	
**C 1*s***	284.6	31.25	284.6	37.59	284.8	63.56	284.7	42.33	Adv. C, C-C, -C=C-
	286.1	42.48	286.1	41.96	286.5	23.77	286.1	41.51	C-N, C-OH, C-O-C, C-Cl
	287.6	20.85	287.4	15.39	287.6	6.46	287.3	12.20	C=O (amide)
	288.9	5.41	288.8	5.06	288.9	6.21	288.9	3.96	Carboxylic
O 1*s*	531.0	30.08	530.9	28.85			531.3	29.21	Carboxylate, C=O (amide)
	532.5	58.31	532.3	56.72	531.6	38.19			C-OH
	533.8	11.61	533.5	14.43					O-H (H_2O)
					532.9	61.81	532.6	70.79	O-P (phosphonic)
N 1*s*	399.2	75.81	399.6	72.09			399.3	46.75	N (amine)
	400.9	24.19					400.6	19.66	N (amide)
					400.1	58.15			N (amine or amide)
			401.77	27.91	402.1	41.85	402.0	33.59	Alkylammonium
P 2*p*					133.8	66.67	132.8	66.67	P $2p_{3/2}$ (phosphonic)
					134.6	33.33	133.7	33.33	P $2p_{1/2}$ (phosphonic)
S 2*p*	167.7	66.67	168.0	48.47	168.1	66.67	168.1	(ε)	S $2p_{3/2}$ (sulfonic)
	168.9	33.33	169.2	24.24	169.3	33.33	169.3	(ε)	S $2p_{1/2}$ (sulfonic)
			163.5	18.2				(ε)	S $2p_{3/2}$ (S-S)
			164.7	9.10				(ε)	S $2p_{1/2}$ (S-S)
Ca $2p_{3/2}$	347.1		347.2		347.5		347.2	(ε)	Ca^{2+}
Cl 2*p*			197.3	11.86		(ε)			Cl $2p_{3/2}$ (Cl)
			198.9	5.93		(ε)			Cl $2p_{1/2}$ (Cl)

	199.9	54.81	(ε)	199.9	66.67	Cl $2p_{3/2}$ (Cl-C covalent bond)
	201.5	27.40	(ε)	201.5	33.33	Cl $2p_{1/2}$ (Cl-C covalent bond)

(ε): weak signal.

Table S7. FTIR assignments and wavenumbers (cm^{-1}) of ALPEI before and after La (III) and Tb (III) (and after 5 cycles of sorption/desorption).

Vibration	Ref.	Wn. in ref.	ALPEI	La(III)		Tb(III)	
				loaded	5 cycles	loaded	5 cycles
**O-H overlapped with N-H stretch**	**[2]**	**3500 -3000**	3402	3392	3404	3397	3405
Aliphatic C-H (stretch)	[2]	2970-2850	2937<br>2775	2926	2933	weak	weak
C=O ester (stretch)	[2]	1750-1725	1753			1751	1753
C=O amide (stretch)/ 1° and 2° amine bend (overlapped)	[1, 4]	1690-1630	1620	1618	1618	1622	1622
C-H/ 1°/2° hydroxyl groups bend and $-COO^-$ salt	[1, 5-7]	1485–1260	1382	1431	1429	1381	1382
C-C, C-O and C-N (stretch)	[2, 14-17]	1350–1000	1033	1033	1033	weak	weak
OH (bend) out-of-plane	[1, 2]	750–590	833	815	817	835	833

Table 8. FTIR assignments and wavenumbers (cm^{-1}) of POH-ALPEI before and after La (III) and Tb(III) (and after 5 cycles of sorption desorption).

Vibration	Ref.	Wn. in ref.	POH-ALPEI	La(III)		Tb(III)	
				loaded	5 cycles	loaded	5 cycles
**O-H overlapped with N-H stretch**	**[2]**	**3500 -3000**	3371	3415	3404	3410	3404
Aliphatic C-H (stretch)	[2]	2970-2850	2939<br>2767	2954	2954	2974	2953
Ester C=O (stretch)	[2]	1750-1725	1753	1701	1699	1703	1703
C=O of amide (stretch)/ 1° and 2° amine bend (overlapped)	[1, 4]	1690-1630	1622	1618	1620	1624	1622
C-H/ 1°/2° hydroxyl groups bend and -COO- salt	[1, 5-7]	1485–1260	1382 (broad)	1381	1383	1384	1383
Asymmetric P=O	[8-11]	1350-1250					
Phosphate ion P(O) (stretch)	[12, 13]	1000-1100	1055	1057	1057	1055	1057
C-C, C-O and C-N (stretch)	[2, 14-17]	1350–1000					
OH (bend) out-of-plane	[1, 2]	750–590					
P-O-C band (stretch)	[11, 18]	570/1100-990	810/570	808/561	810/567	808/563	808/563
Sulfate ion	[1, 19]	680–610		617	617		

Table 9. Assignments and interpretation of major XPS signals for POH-A$_L$PEI before and after La (III) and Tb (III) sorption.

Signal	POH-A$_L$PEI		Sorbent + La (III)		Sorbent + Tb (III)		Assignment
	BE (eV)	AF (%)	BE (eV)	AF (%)	BE (eV)	AF (%)	
C 1*s*	284.7	42.33	284.5	33.09	284.7	37.90	Adv. C, C-C, -C=C-
	286.1	41.51	286.0	44.71	286.1	40.87	C-N, C-OH, C-O-C
	287.3	12.20	287.3	14.75	287.4	14.34	C=O (amide)
	288.9	3.96	288.6	7.45	288.7	6.89	Carboxylic
O 1*s*	531.3	29.21	530.6	15.54	530.9	11.76	Carboxylate, C=O (amide)
	532.6	70.79	532.2	69.21	532.4	63.00	O-P (phosphonic)
			533.8	15.25	533.7	25.25	O-H (H_2O)
N 1*s*	399.3	46.75	399.6	57.76	398.4	59.23	N (amine)
	400.6	19.66	401.3	31.54	400.3	26.65	N (amide)
	402.0	33.59	402.4	10.71	401.3	14.13	Alkylammonium
P 2*p*	132.8	66.67	134.4	66.67	133.3	66.67	P $2p_{3/2}$ (phosphonic)
	133.7	33.33	135.2	33.33	134.1	33.33	P $2p_{1/2}$ (phosphonic)
S 2*p*	168.1	(ε)	167.9	66.67	167.9	66.67	S $2p_{3/2}$ (sulfonic, sulfate)
	169.3	(ε)	169.1	33.33	169.1	33.33	S $2p_{1/2}$ (sulfonic, sulfate)
Ca 2p	347.2	(ε)					Ca $2p_{3/2}$
Cl 2*p*	199.9	66.67					Cl $2p_{3/2}$ (Cl-C covalent bond)
	201.5	33.33					Cl $2p_{1/2}$ (Cl-C covalent bond)
La 3d			~ 835[(a)]				La $3d_{5/2}$ (La(III))
			~ 851[(a)]				La $3d_{3/2}$ (La(III))
Tb 4d					152-151		Tb $4d_{5/2}$ (Tb(IV))
					~ 149 (ε)		Tb $4d_{5/2}$ (Tb(III))

[(a)] Multiplets with ΔBEs ~ 3.2-3.4 eV

Table S10. SEM micrographs and semi-quantitative EDX analysis of surface of POH-A$_L$PEI after La (III) and Tb (III) sorption at pH$_0$ 5.

Sorbed metal ion	SEM	EDX analysis
La (III)	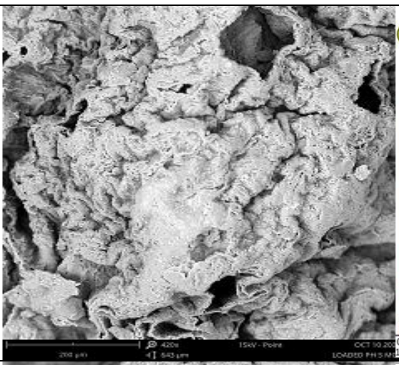	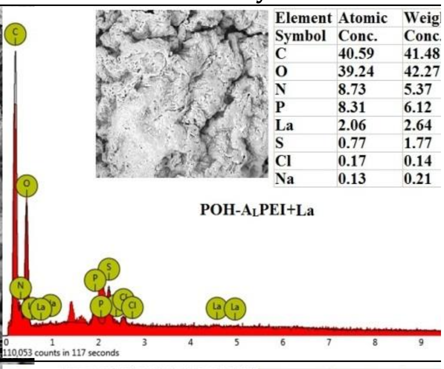
Tb(III)	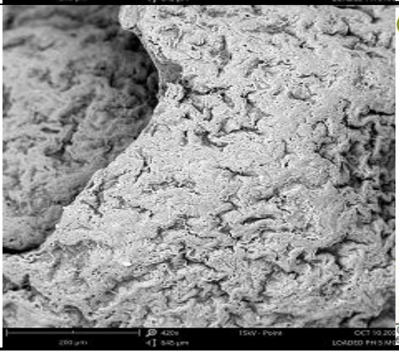	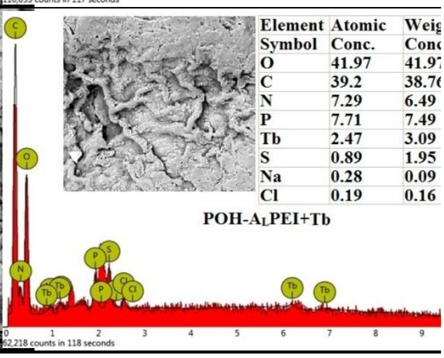

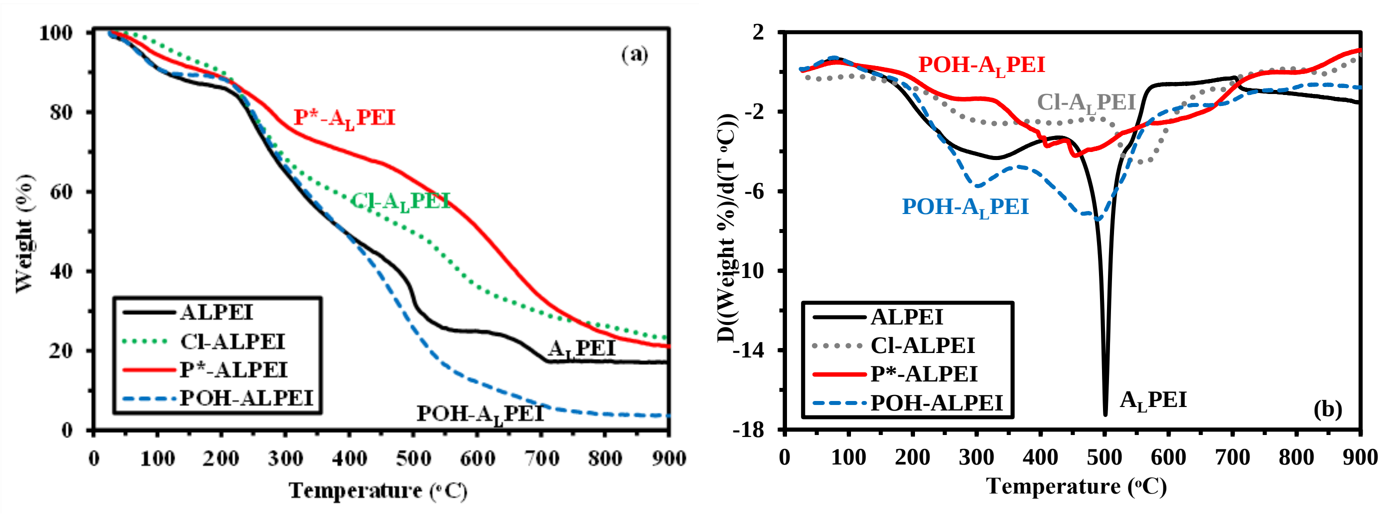

Figure 1. Thermal degradation of A$_L$PEI beads, intermediary products (epichlorohydrine-activated A$_L$PEI, Cl-A$_L$PEI, and phosphorylated A$_L$PEI beads, P*-A$_L$PEI), and sorbent (de-esterified phosphorylated beads, POH-A$_L$PEI): (a) TGA, and (b) DrTG.

Material	Transition Range (°C)	Extrema DrTG (°C)	Weight loss (%)	Total weight Loss (%)
A$_L$PEI	27.81-197.81		13.74	82.87
	197.81-481.01	319.09	46.90	
	481.01-638.81	500.21	15.38	
	638.81-909.41	696.61	6.85	
Cl-A$_L$PEI	36.01-195.01	58.01	9.40	76.65
	195.01-497.81	301.01-406.41	40.63	
	497.81-909.61	530.41-549.01-674.77-836.02	26.62	
P*-APEI	27.63-227.63		13.78	78.94
	227.63-445.43	253.83-406.8	18.83	
	445.43-909.23	449.02-792.63	46.33	
POH-A$_L$PEI	24.86-194.86		11.35	96.24
	194.86-549.26	290.06-455.26-480.2	72.16	
	549.26-909.26	671.06-768.5	12.72	

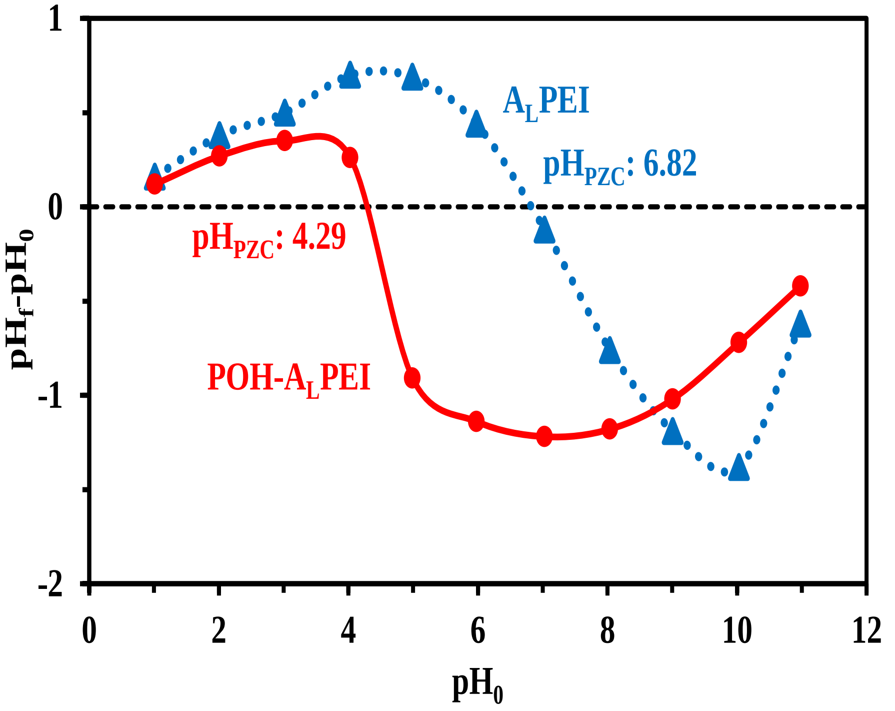

Figure S2. Determination of the pH$_{PZC}$ of A$_L$PEI and POH-A$_L$PEI sorbents using the pH drift method (Sorbent dosage, SD: 2 g L^{-1}; time: 48 h; background salt: 0.1 M NaCl).

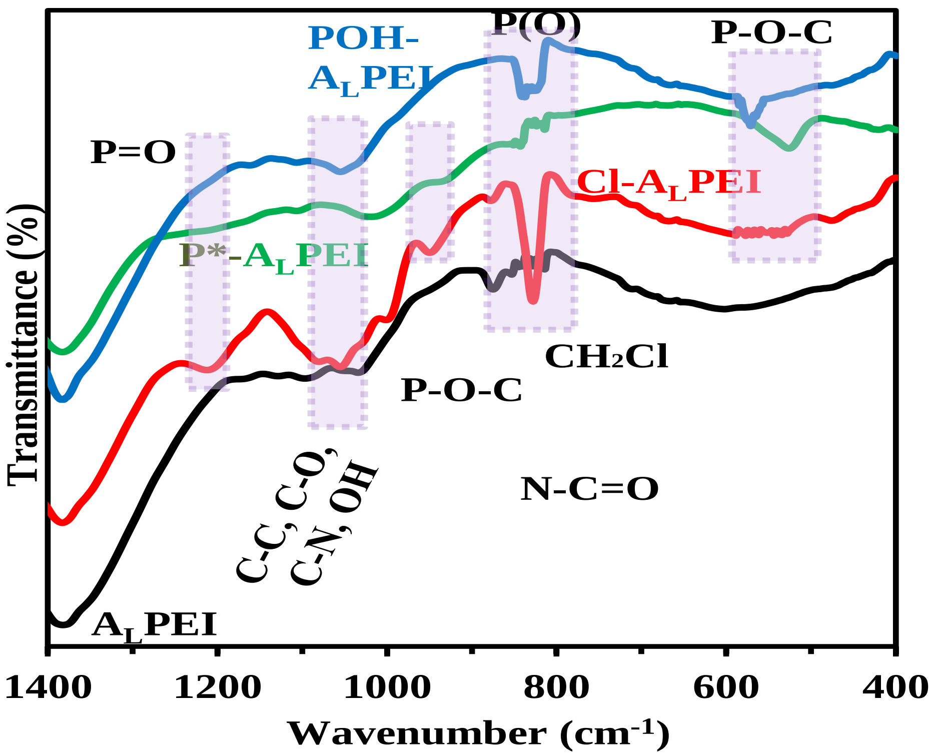

Figure S3. Focused FTIR spectra of A$_L$PEI, POH-A$_L$PEI and intermediary products.

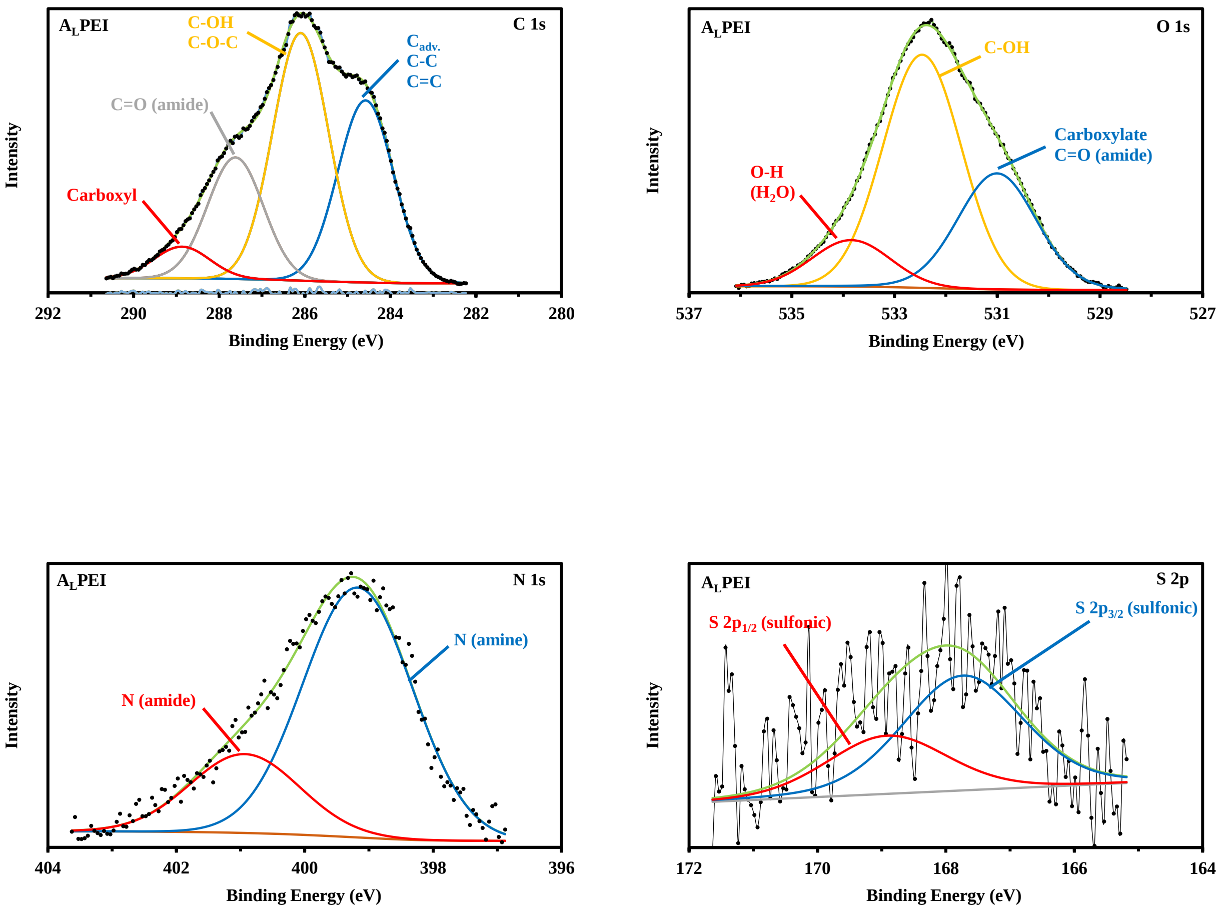

Figure 4. HRES XPS C 1*s*, O 1*s*, N 1*s* and S 2*p* core level spectra for ALPEI.

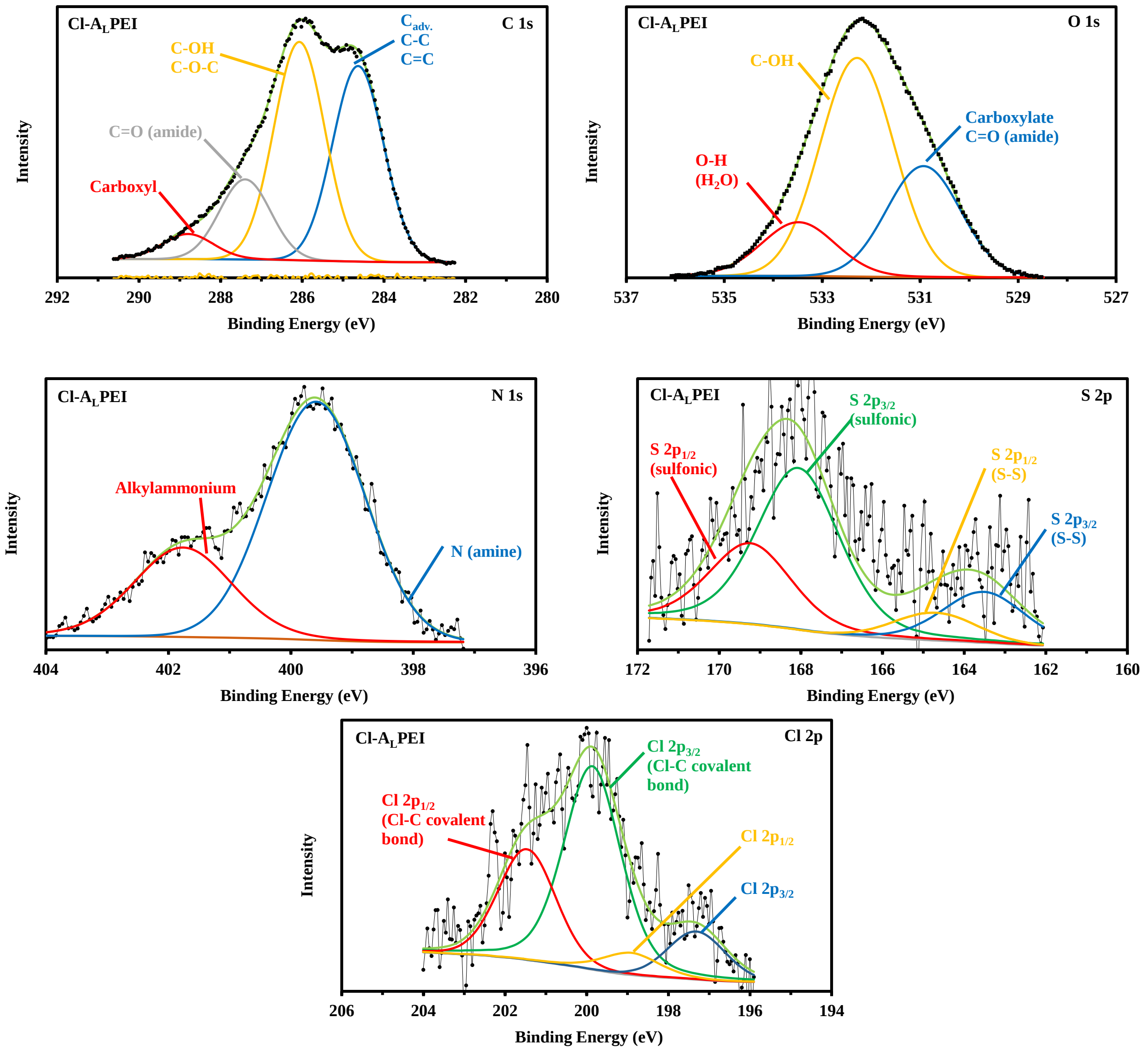

Figure 5. HRES XPS C 1*s*, O 1*s*, N 1*s*, S 2*p* and Cl 2*p* core level spectra for Cl-ALPEI.

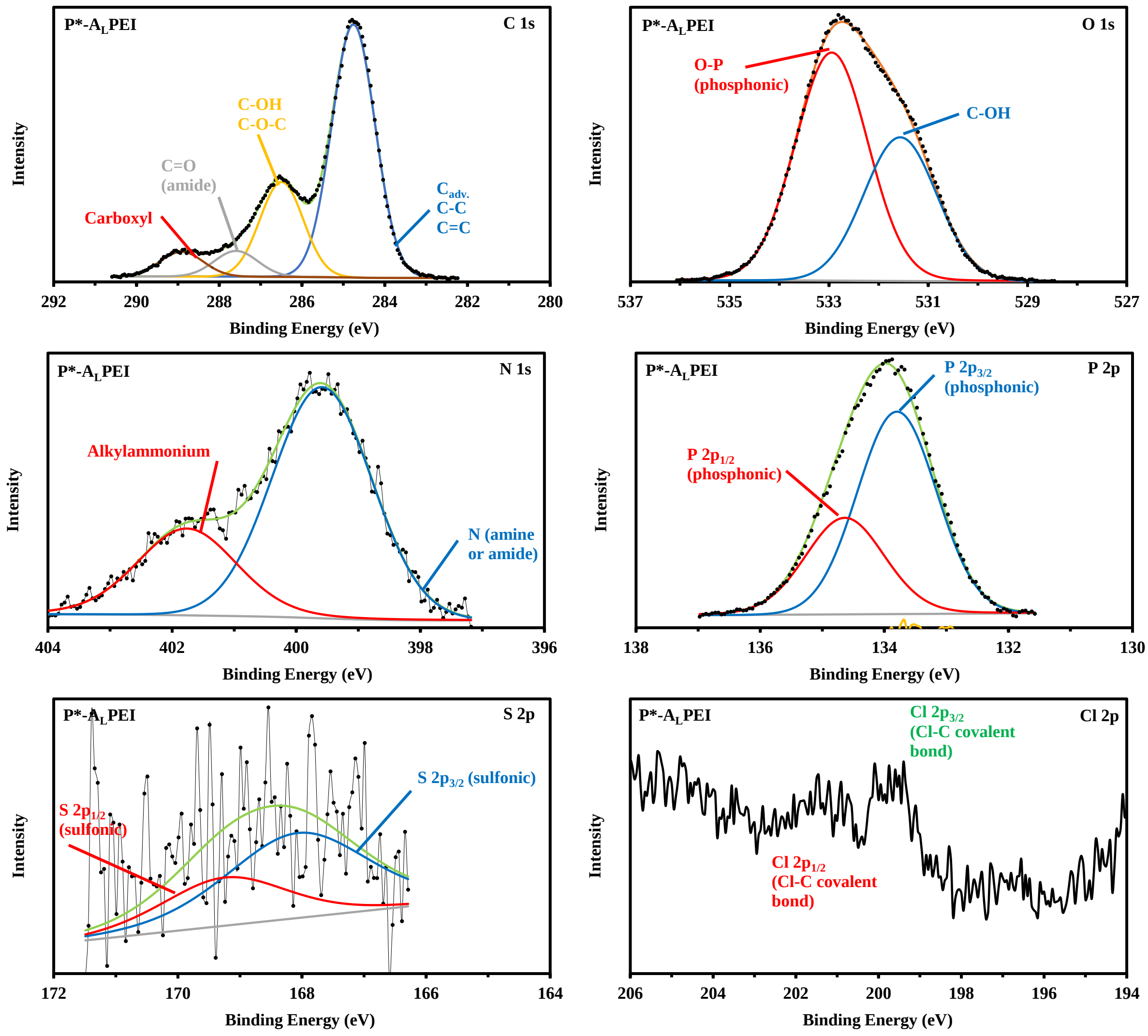

Figure S6. HRES XPS C 1*s*, O 1*s*, N 1*s*, P 2*p*, S 2*p* and Cl 2*p* core level spectra for POH-A$_L$PEI.

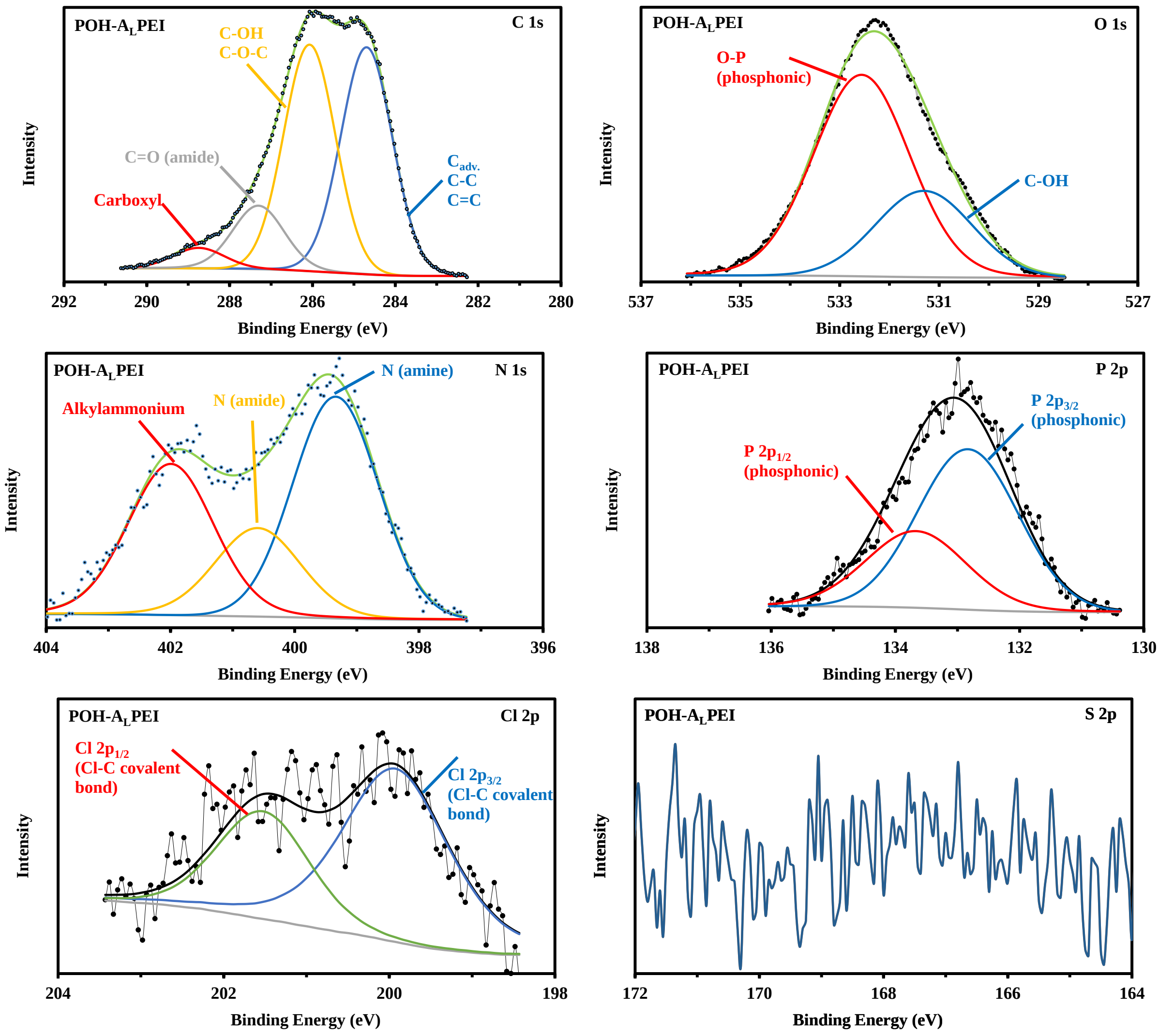

Figure 7. HRES XPS C 1*s*, O 1*s*, N 1*s*, P 2*p*, Cl 2*p* and S 2*p* core level spectra for POH-A$_L$PEI.

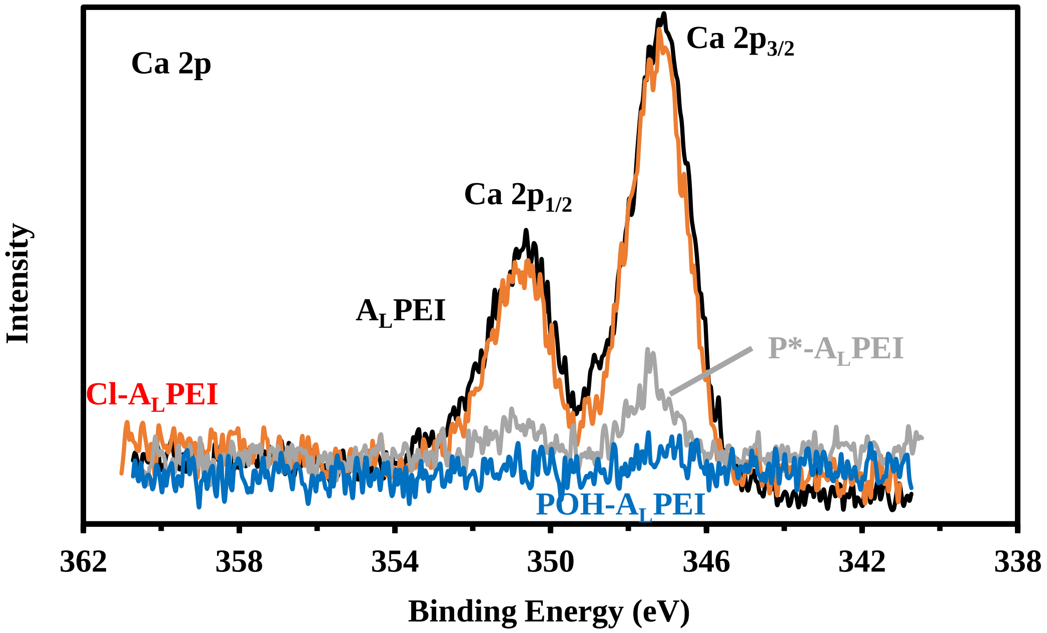

Figure 8. HRES XPS Ca 2*p* core level spectra for ALPEI, Cl-ALPEI, P*-ALPEI and POH-ALPEI.

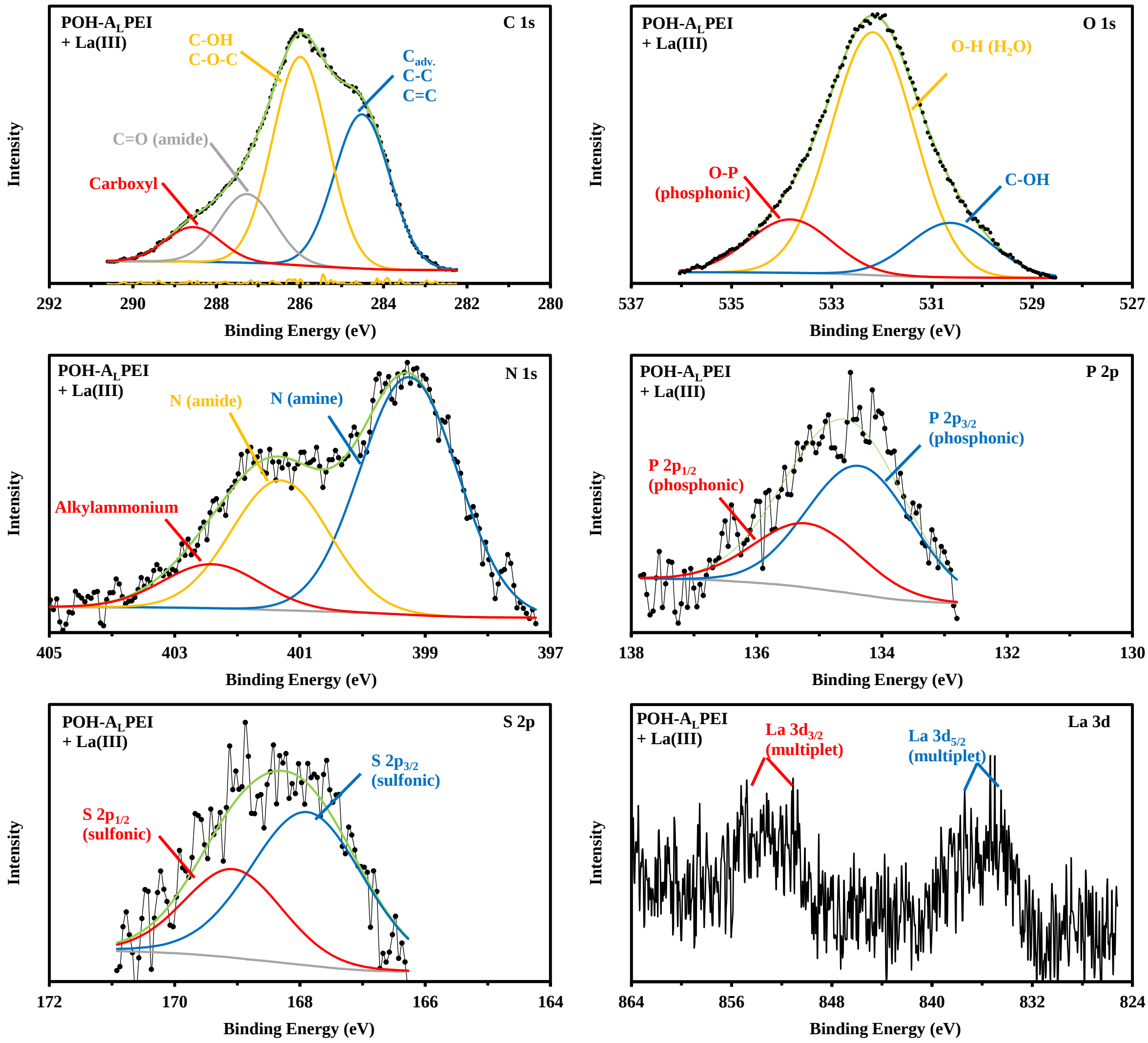

Figure 9. HRES XPS C 1*s*, O 1*s*, N 1*s*, P 2*p*, S 2*p* and La 3*d* core level spectra for POH-ALPEI after La (III) sorption.

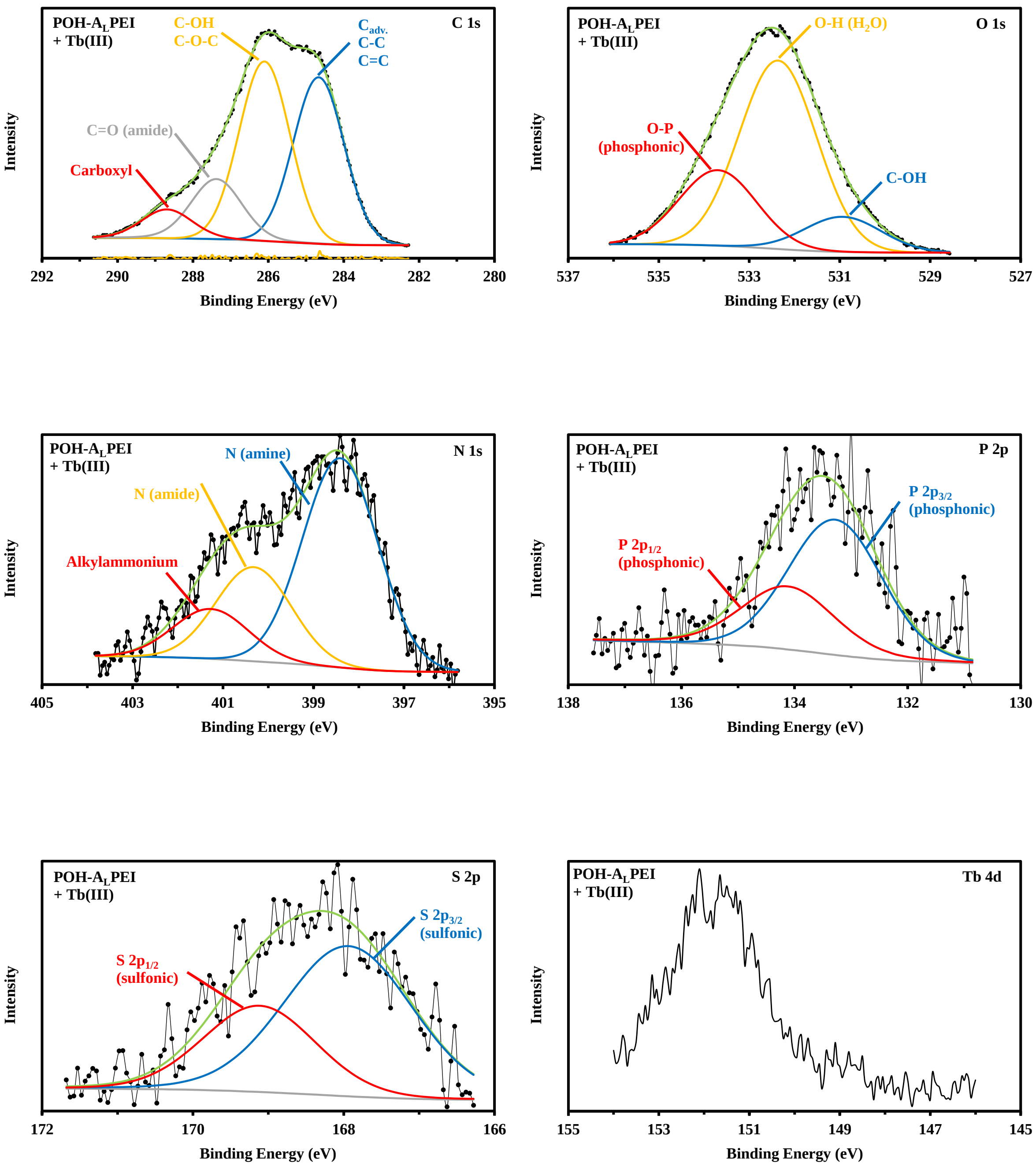

Figure 10. HRES XPS C 1*s*, O 1*s*, N 1*s*, P 2*p*, S 2*p* and Tb 4*d* core level spectra for POH-ALPEI after Tb (III) sorption.

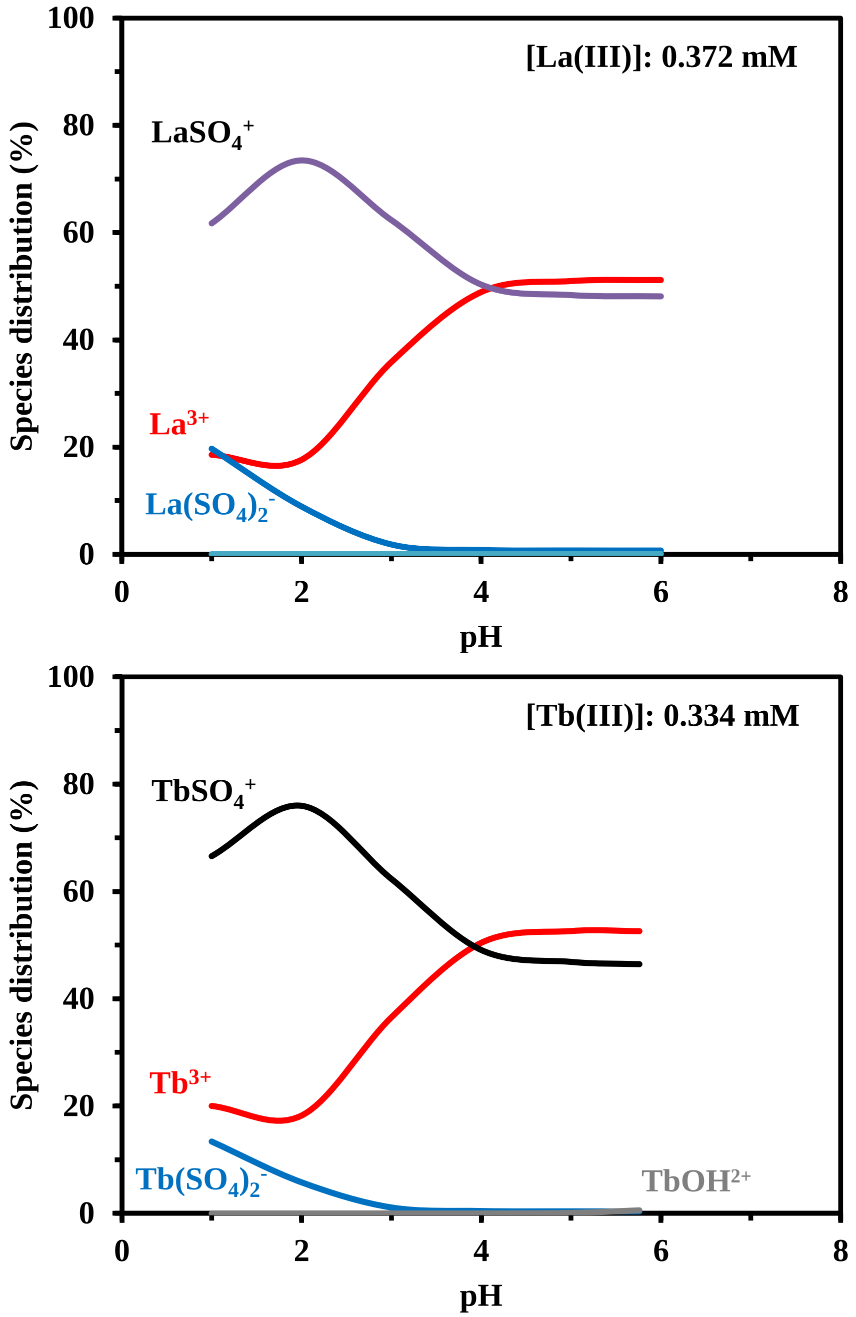

Figure 11. Speciation diagrams for La (III) and Tb (III) (under experimental conditions corresponding to the study of pH effect: C_0: 0.372 mmol La L^{-1} and 0.334 mmol Tb L^{-1}).

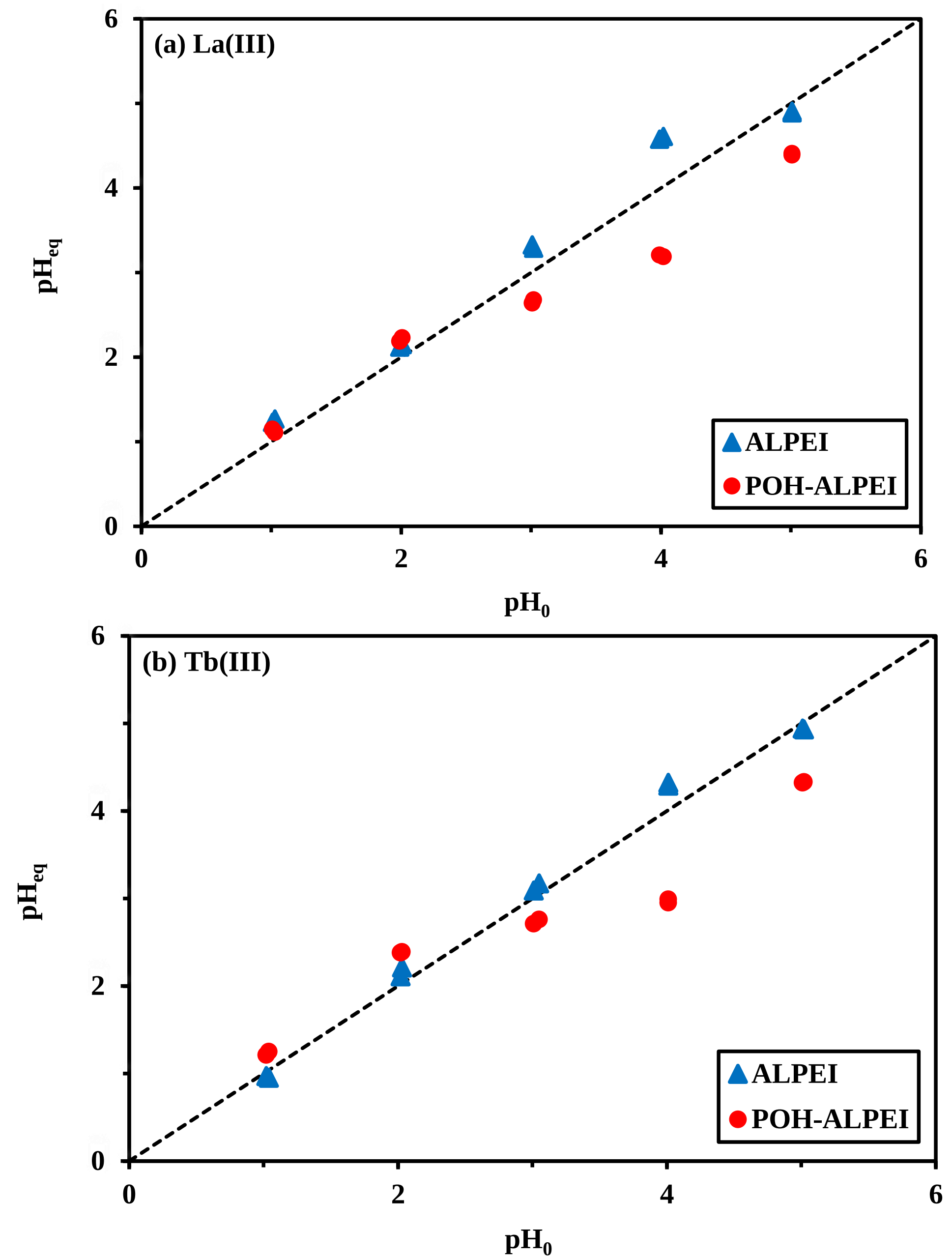

Figure 12. pH variation during La (III) (a) and Tb (III) (b) sorption using ALPEI and POH-ALPEI sorbents (C_0: 50 mg L^{-1} = 0.374 mmol La L^{-1} or 0.332 mmol Tb L^{-1}; Sorbent dosage, SD: 0.66 g L^{-1}; time: 48 h; T: 21 ±1 °C).

References

1. Coates, J., Interpretation of Infrared Spectra, A Practical Approach. In *Encyclopedia of Analytical Chemistry*, Meyers, R. A., Ed. John Wiley & Sons Ltd: Chichester, U.K., 2000; pp 10815-10837.
2. Coates, J., Interpretation of Infrared Spectra, A Practical Approach. In *Encyclopedia of Analytical Chemistry* John Wiley & Sons, Ltd.: 2006; pp 1-23.
3. Hamza, M. F.; Abdel-Rahman, A. A. H.; Guibal, E., Magnetic glutamine-grafted polymer for the sorption of U(VI), Nd(III) and Dy(III). *J. Chem. Technol. Biotechnol.* **2018,** 93, (6), 1790-1806.
4. Mohammadi, N.; Ganesan, A.; Chantler, C. T.; Wang, F., Differentiation of ferrocene D-5d and D-5h conformers using IR spectroscopy. *J. Organomet. Chem.* **2012,** 713, 51-59.
5. Chen, Y.; Geng, J.; Zhuang, Y.; Zhao, J.; Chu, L.; Luo, X.; Zhao, Y.; Guo, Y., Preparation of the chitosan grafted poly (quaternary ammonium)/Fe_3O_4 nanoparticles and its adsorption performance for food yellow 3. *Carbohydr. Polym.* **2016,** 152, 327-336.
6. Colthup, N. B.; Daly, L. H.; Wiberley, S. E., *Introduction to Infrared and Raman Spectroscopy*. 3rd. ed. ed.; Academic Press, Inc.: San Diego, CA (USA), 1990; p 560.

7. Hu, X. J.; Wang, J. S.; Liu, Y. G.; Li, X.; Zeng, G. M.; Bao, Z. L.; Zeng, X. X.; Chen, A. W.; Long, F., Adsorption of chromium (VI) by ethylenediamine-modified cross-linked magnetic chitosan resin: Isotherms, kinetics and thermodynamics. *J. Hazard. Mater.* **2011,** 185, (1), 306-314.
8. Benning, L. G.; Phoenix, V. R.; Yee, N.; Tobin, M. J., Molecular characterization of cyanobacterial silicification using synchrotron infrared micro-spectroscopy. *Geochim. Cosmochim. Acta* **2004,** 68, (4), 729-741.
9. Dean, A. P.; Martin, M. C.; Sigee, D. C., Resolution of codominant phytoplankton species in a eutrophic lake using synchrotron-based Fourier transform infrared spectroscopy. *Phycologia* **2007,** 46, (2), 151-159.
10. Giordano, M.; Kansiz, M.; Heraud, P.; Beardall, J.; Wood, B.; McNaughton, D., Fourier transform infrared spectroscopy as a novel tool to investigate changes in intracellular macromolecular pools in the marine microalga *Chaetoceros muellerii* (Bacillariophyceae). *J. Phycol.* **2001,** 37, (2), 271-279.
11. Nandiyanto, A. B. D.; Oktiani, R.; Ragadhita, R., How to read and interpret FTIR spectroscope of organic material. *Indones. J. Sci. Technol.* **2019,** 4, (1), 97-118.
12. Cochez, M.; Ferriol, M.; Weber, J. V.; Chaudron, P.; Oget, N.; Mieloszynski, J. L., Thermal degradation of methyl. methacrylate polymers functionalized by phosphorus-containing molecules - I. TGA/FT-IR experiments on polymers with the monomeric formula $CH_2C(CH_3)C(O)OCHRP(O)(OC_2H_5)_2$ (R=H, $(CH_2)_4CH_3$, C_6H_5Br, $C_{10}H_7$). *Polym. Degrad. Stab.* **2000,** 70, (3), 455-462.
13. Wei, Y.; Salih, K. A. M.; Rabie, K.; Elwakeel, K. Z.; Zayed, Y. E.; Hamza, M. F.; Guibal, E., Development of phosphoryl-functionalized algal-PEI beads for the sorption of Nd(III) and Mo(VI) from aqueous solutions – Application for rare earth recovery from acid leachates. *Chem. Eng. J.* **2021**, Art. N° 127399.
14. Zhao, Z.; Xie, X.; Wang, Z.; Tao, Y.; Niu, X.; Huang, X.; Liu, L.; Li, Z., Immobilization of *Lactobacillus rhamnosus* in mesoporous silica-based material: An efficiency continuous cell-recycle fermentation system for lactic acid production. *J. Biosci. Bioeng* **2016,** 121, (6), 645-651.
15. Namdeo, M.; Bajpai, S. K., Chitosan-magnetite nanocomposites (CMNs) as magnetic carrier particles for removal of Fe(III) from aqueous solutions. *Colloids Surf., A* **2008,** 320, (1-3), 161-168.
16. Oshita, K.; Takayanagi, T.; Oshima, M.; Motomizu, S., Adsorption behavior of cationic and anionic species on chitosan resins possessing amino acid moieties. *Anal. Sci.* **2007,** 23, (12), 1431-1434.
17. Zhang, X.; Jiao, C.; Wang, J.; Liu, Q.; Li, R.; Yang, P.; Zhang, M., Removal of uranium(VI) from aqueous solutions by magnetic Schiff base: Kinetic and thermodynamic investigation. *Chem. Eng. J.* **2012,** 198, 412-419.
18. Hamza, M. F.; Roux, J.-C.; Guibal, E., Uranium and europium sorption on amidoxime-functionalized magnetic chitosan micro-particles. *Chem. Eng. J.* **2018,** 344, 124-137.
19. Sifontes, A. B.; Canizales, E.; Toro-Mendoza, J.; Avila, E.; Hernandez, P.; Delgado, B. A.; Brenda Gutierrez, G.; Diaz, Y.; Cruz-Barrios, E., Obtaining highly crystalline barium sulphate nanoparticles via chemical precipitation and quenching in absence of polymer stabilizers. *J. Nanomater.* **2015,** 2015, Art. N° 510376.
